# Experimental Study on the Seismic Performance of Recycled Concrete Brick Walls Embedded with Vertical Reinforcement

**DOI:** 10.3390/ma7085934

**Published:** 2014-08-19

**Authors:** Wanlin Cao, Yongbo Zhang, Hongying Dong, Zhongyi Zhou, Qiyun Qiao

**Affiliations:** College of Architecture and Civil Engineering, Beijing University of Technology, Beijing 100124, China; E-Mails: wlcao@bjut.edu.cn (W.C.); zhangyongbo@emails.bjut.edu.cn (Y.Z.); zhouzy@bjut.edu.cn (Z.Z.); qiaoqiyun@bjut.edu.cn (Q.Q.)

**Keywords:** recycled concrete brick (RCB), compressive performance, seismic performance, experimental study, numerical simulation

## Abstract

Recycled concrete brick (RCB) is manufactured by recycled aggregate processed from discarded concrete blocks arising from the demolishing of existing buildings. This paper presents research on the seismic performance of RCB masonry walls to assess the applicability of RCB for use in rural low-rise constructions. The seismic performance of a masonry wall is closely related to the vertical load applied to the wall. Thus, the compressive performance of RCB masonry was investigated firstly by constructing and testing eighteen RCB masonry compressive specimens with different mortar strengths. The load-bearing capacity, deformation and failure characteristic were analyzed, as well. Then, a quasi-static test was carried out to study the seismic behavior of RCB walls by eight RCB masonry walls subjected to an axial compressive load and a reversed cyclic lateral load. Based on the test results, equations for predicting the compressive strength of RCB masonry and the lateral ultimate strength of an RCB masonry wall were proposed. Experimental values were found to be in good agreement with the predicted values. Meanwhile, finite element analysis (FEA) and parametric analysis of the RCB walls were carried out using ABAQUS software. The elastic-plastic deformation characteristics and the lateral load-displacement relations were studied.

## 1. Introduction

China is a large resource consumer because of the rapid growth of its economics and environmental issues. According to statistics, the consumption of cement in China is 820 million tons and accounts for 55% of the world’s consumption. It is estimated that about 200 million tons of waste concrete are produced each year in the mainland of China [1]. Recycling and reusing construction waste has become one of the key issues that should be solved urgently in China with the rapid urbanization, new countryside construction and large-scale urban renewal. In view of environmental protection, the conservation of natural resources and the shortage of lands for waste disposal, traditional clay brick has been forbidden by China’s government. There is thus an urgent need to develop new brick suitable for residential buildings in view of masonry structures that are still playing a significant role in rural low-rise constructions. The difficulties of the disposal of discarded concrete blocks and demolition waste with a developing scarcity of virgin aggregate have appealed to the use of recycled aggregate instead of natural aggregate. Crushed concrete rubble, after separation from other waste and being sieved, can be used as a substitute for natural coarse aggregates in concrete or as a sub-base or a base layer in pavements [2,3,4,5]. This type of recycled material is called recycled aggregate. Recycled aggregate can roughly be divided into two categories, namely recycled coarse aggregate and recycled fine aggregate. The recycled concrete bricks molded with recycled aggregate are suitable for rural low-rise constructions.

Previous research has focused on the material performance, mechanics performance and seismic performance of recycled aggregate concrete structures and has accumulated many achievements. The research of recycled concrete brick has been little studied, especially the seismic design of an RCB wall structure in rural low-rise constructions. In fact, an attempt has been made by Collins *et al*. [6] to use recycled aggregates in making blocks for a beam-and-block floor system. The blocks were 440 mm × 215 mm × 100 mm in dimension and were produced at a block factory. Recycled aggregates were used to replace 25%–75% by weight of the natural aggregate (including coarse and fine aggregates). A compressive strength of 6.75 MPa and a transverse strength of 1.23 MPa were reported for the blocks, with 75% of the natural aggregates replaced by recycled aggregates. Poon *et* *al*. [7] studied the applicability of recycled aggregates in molded recycled concrete bricks and blocks by their compressive and bending performance test, including their compressive strength, transverse strength, shrinkage, skid resistance and density. Results showed that the replacement of coarse and fine natural aggregates by recycled aggregates at the levels of 25% and 50% had little effect on the compressive strength of the bricks and blocks, but higher levels of replacement reduced the compressive strength. The compressive strength of recycled concrete brick decreased when the recycled aggregate substitution ratio exceeded 50%. Gu *et al.* [8] tested the mechanical performance of recycled concrete brick walls under repeated loading and analyzed the bearing capacity and failure process of recycled concrete brick walls. Cao *et al.* [9] tested the seismic performance of the RCB masonry structure by the shake table test of two RCB masonry buildings with or without vertical reinforcement. Results have shown that an RCB masonry building with vertical reinforcement exhibited better seismic performance and a lighter degree of damage than that of the ordinary RCB masonry building. He *et al.* [10] studied the compressive and bending performance of RCB with a compressive strength range from 15 to 30 MPa. The impact of recycled aggregate on the solid concrete brick mechanical properties and physical properties was analyzed. Guo *et al.* [11] investigated the seismic performance of recycled concrete perforated brick masonry subjected to low frequency reversed cyclic load. The test results show that the seismic performance of recycled concrete perforated brick masonry is similar to those of ordinary concrete perforated brick walls with good hysteresis loops and ductility, as well as strong energy dissipation capacities. He *et al.* [12] studied the drying shrinkage performance of solid concrete bricks made from recycled coarse aggregate. Results showed that recycled aggregate can increase the shrinkage of solid concrete brick. The shrinkage of recycled solid concrete bricks (fully substituted) is larger than ordinary concrete bricks by 1.6–2 times. A water reducer is helpful to improve the water retention and decrease the shrinkage of brick. Valdés *et al*. [13] investigated the physical and mechanical properties of concrete bricks produced with recycled aggregates. The results show that the compressive strength of the concrete bricks manufactured with recycled material showed resistances of 15% lower than those manufactured with natural aggregates. In view of the shortcomings of previous studies, it is worthwhile to investigate the seismic performance of RCB masonry walls in order to prompt a better application in rural low-rise constructions.

It has been known that the seismic performance of masonry walls is closely related to the vertical load applied on the wall. So far, a variety of studies has been carried out to study the shear-compression ratios and the failure criterion of masonry. These studies were all based on common brick masonry without reinforcement. Lourenco *et al*. [14] analyzed the horizontal force and horizontal displacement relations, and failure criterion expressions were proposed, as well. It is stated that the failure model of masonry can be divided into two types: Tension-controlled failure and compression-controlled failure. The proposed failure criterion expressions cannot explain the failure that may occur in a masonry structure. The failure criterion that is proposed by Andreaus [15] can explain all of the failure patterns of masonry, including tension-control, compression-control and sliding-control failure; however, it was too complex to implement. Shi [16] pointed out that the failure models of a masonry wall depended on the size of the vertical load and can be classified as shear-friction, shear-compression and diagonal compression. Luo *et al*. [17] tested the shear performance of masonry and achieved shear-compression correlation curves. Studies have shown that the shear strength of masonry increased with the axial stress ratio increasing in the range of 0–0.6, but the increasing gradient becomes smaller. When the axial stress ratio exceeded 0.6, the shear strength of masonry dropped rapidly and, then, down to zero when the axial stress ratio reached 1.0. The shear-compression correlation curve of masonry proposed by Cai *et al*. [18] showed that the shear strength of masonry reached the maximum value when the axial stress ratio was equal to 0.475. Liu *et al*. [19] established the failure criterion of masonry in shear-compression on the basis of mechanic principles and a statistical method. A calculation method of shear strength by the failure criterion was proposed, as well. It pointed out that the axial stress ratio of 0.32 was the boundaries between shear sliding failure and tensile failure and the axial stress ratio of 0.67 was between tensile failure and compression failure. Therefore, to study the seismic performance of RCB masonry walls, we first need to explore the compressive performance of RCB masonry.

To investigate the applicability of RCB masonry in rural low-rise constructions extensively, two critical technical problems of RCB masonry were investigated, the compressive performance and the seismic performance. The compressive performance was studied first based on eighteen RCB compressive specimens with different mortar strengths under vertical load. The compressive strength, deformation and failure characteristic were analyzed. Then, the seismic performance was investigated based on eight RCB masonry walls with different parameters under reversed cyclic lateral loads. Their seismic performance was comparatively analyzed. Furthermore, an elastic-plastic finite element analysis (FEM) and parametric analysis of the RCB masonry walls were carried out by ABAQUS software. The elastic-plastic deformation characteristics and the relationships of lateral load to top displacement were studied. The proposed approach, which is named recycled concrete brick walls embedded with vertical reinforcement, has had a national invention patent applied for and authorized. The paper aims to investigate its superiority to traditional approaches that are accepted internationally and to explore its applicability in rural areas.

## 2. Experimental Section

### 2.1. Compressive Performance of RCB Masonry

#### 2.1.1. Materials and Specimen Preparation

The RCB was manufactured by a mechanized molding machine in Beijing (YuanTaiDa Environmental Protection Building Materials Technology Limited Liability Company of China). Table 1 shows the mix proportions for recycled concrete bricks. The cementitious materials used were an ordinary Portland cement (P·O 42.5) complying with GB175-2007 [20]. The recycled aggregates were C&D (Construction and Demolition) wastes sourced from a demolished house in Beijing. Then, the C&D wastes underwent a further process of mechanized crushing and sieving to produce fine aggregate and coarse aggregate in the factory mentioned above. The marl, containing some clay, was a kind of precipitate particle obtained from the sedimentation tank after the recycled aggregates were rinsed by water. The fly ash was a kind of fine powder, which was collected by the aspiration air separation system during the production process of the recycled aggregate. The mixed materials are molded under a combined vibrating and compacting action. Then, the bricks were removed from the molds and cured in air at room temperature for 28 days. The measured average compressive strength of 28 days for the RCB was 7.9 MPa.

**Table 1:** Mix proportions for recycled concrete bricks. RCB: recycled concrete brick.

Mix Notation	Proportion (kg)				
	Cement	Recycled Fine Aggregate	Marl	Fly Ash	Added Water
RCB	143	608	152	240	120

**Figure 1:**
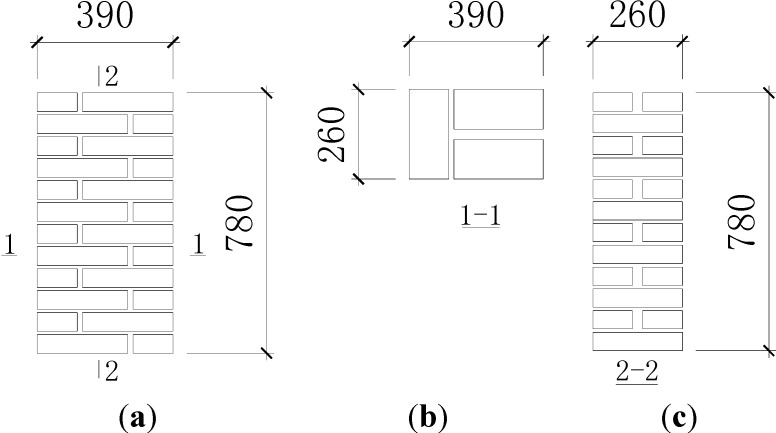
The section details of the compressive specimen: (**a**) facade size; (**b**) Section 1-1; (**c**) Section 2-2. (Unit: millimeters).

Eighteen RCB masonry compressive specimens with different mortar strengths were designed and tested under a vertical load. The specimens contained three groups, which were called A, B and C, respectively, corresponding to the designed mortar strength of M5, M7.5 and M10. Each group contained six specimens. KYM 5-1–KYM 5-6 were designed with a mortar strength of 5 MPa in Group A. KYM 7.5-1–KYM 7.5-6 were designed with a mortar strength of 7.5 MPa in Group B, and KYM 10-1–KYM 10-6 were designed with a mortar strength of 10 MPa in Group C (KYM stand for the compressive masonry in Chinese). The dimensions of all of the RCB masonry specimens were 780 mm × 260 mm × 390 mm. The dimensions of the RCB brick were 53 mm × 115 mm × 260 mm. The measured average mortar strengths of 28 days for Groups A–C were 4.84, 7.33 and 9.44 MPa, respectively. The details of the compressive specimen are shown in Figure 1.

The compressive strength of masonry is affected by certain factors, such as the size and shape of the masonry, the loading method, and so on. The cross-section size of standard brick masonry specimen adopted by the former Soviet Union is 370 mm × 490 mm. RILEM (International Union of Laboratories and Experts in Construction Materials, Systems and Structures) recommends a one-story-height brick wall or brick column as a standard brick masonry specimen. The United States, Canada and Australia adopt a prism piled up by laying bricks or stones as the brick masonry standard specimen. While in China, the standard cross-section size of brick masonry is 240 mm × 370 mm, the ratio of the masonry height to the short length of the cross-section is smaller than 2.5–3.0. This results in determining the current specification of brick masonry compression strength. The strength conversion coefficient is calculated by Equation (1) [16] when the brick masonry has a non-standard cross section.

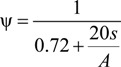
(1)
where ψ is the strength conversion coefficient, *S* is the perimeter of the non-standard cross-section and *A* is the area of the non-standard cross-section. Thus, considering the RCB size, we adopted the cross-section of the specimens by 780 mm × 260 mm × 390 mm.

#### 2.1.2. Test Setup and Loading Procedure

**Figure 2:**
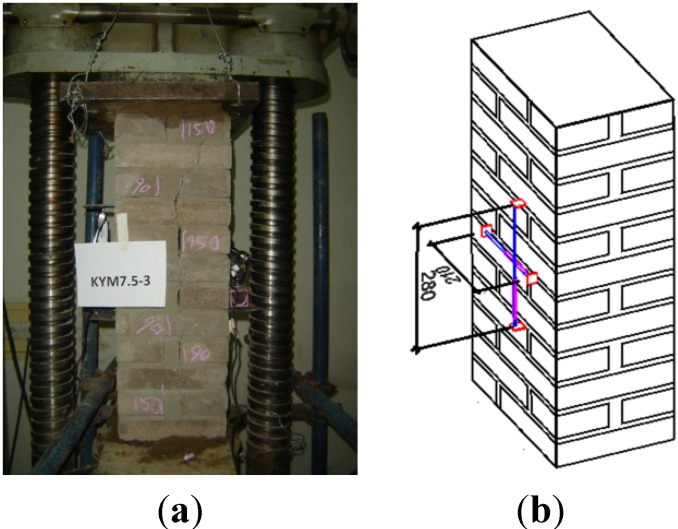
(**a**) Test set-up; (**b**) Dial indicator arrangement.

The specimens were tested according to the standard test method of the basic mechanics properties of masonry (GB/T 50129-2011) [21]. The vertical load was applied by steps, and the value of each step was 10% of the predicted failure load. The load of each step was applied completely within 1–1.5 min and maintained for 1–2 min before the next step. When the total load value reached 80% of the predicted failure load, continuous load was applied with the original load speed until the specimens were damaged. The maximum load value, which is read from the testing machine, was the failure load of the specimen. Dial indicators were set at the two long sides of the specimen to monitor the axial compressive and lateral expansive deformation, with a gauge length of 280 mm and 210 mm, respectively. The test setup and dial indicator arrangement are illustrated in Figure 2.

#### 2.1.3. Failure Characteristics

The failure process of RCB masonry contained two stages. The first stage was from the start to the occurrence of the first crack. With the increase of the load, the first crack appeared in the vertical mortar joint at the top of the short edge. The second stage was from crack to failure. With the increase of the load, cracks at the short edge developed downwards along the vertical mortar joint and penetrated at the lower part of the masonry gradually. Meanwhile, vertical cracks also appeared in the center of the long edge and developed gradually, until the specimens cracked.

**Figure 3:**
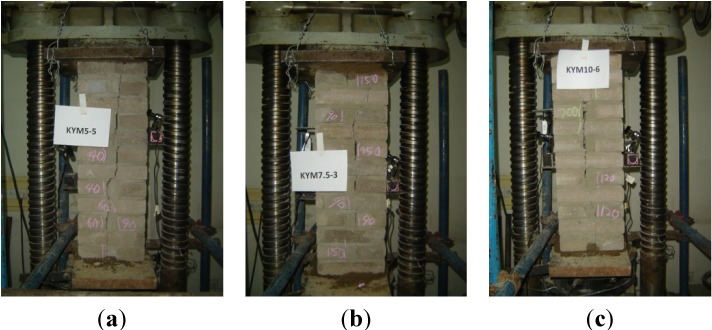
Typical failure characteristics: (**a**) KYM5-5; (**b**) KYM7.5-3; (**c**) KYM10-6.

The failure process of the three groups of specimens is as follows: (1) Six specimens of Group A: Due to the setting deviation and asymmetry load, the corner of KYM5-1 crushed first, because of compression, and then, the specimen cracked; as for the remaining five specimens, the first crack appeared in the vertical mortar joint at the top of the short edge, then vertical cracks also appeared at the long edge. With the increase of the load, cracks developed gradually until they were through the wall, and finally, the specimens cracked; (2) Six specimens of Group B: As for specimen KYM7.5-4, the first crack appeared in the vertical mortar joint in the middle of the short edge and developed rapidly as the load increased, its top corner crushed, then the specimen cracked; as for the other five specimens, the failure process was similar to the specimens of Group A, the difference being that the bearing capacity increased with the improvement of the mortar strength; (3) Six specimens of Group C: The failure process was also similar to specimens of Groups A and B; the bearing capacity was higher, because of the higher mortar strength. The typical failure characteristics of these three group specimens are shown in Figure 3.

#### 2.1.4. Measured Compressive Strength

The measured cracking load, failure load and compressive strength of the specimens are listed in Table 2, where *P*_c_ is the cracking load, *P*_u_ is the failure load and *f*_c,m_ is the average compressive strength. In the specimen named KYM *i-j*, KYM stand for the compressive masonry in Chinese; *i* represents the designed mortar strength grade of each group, namely, *i* equals 5, 7.5 and 10, representing Groups A, B and C, respectively. *j* represents the number of the specimens in each group. It points out that three specimens of group C were cured outdoors in the winter due to the space constraints and were damaged for a certain reason, while other specimens were cured indoors. Thus, the first 3 results for Group C are missing from Table 2. It can be seen from Table 2 that the cracking load and failure load of RCB masonry increased as the mortar strength increased. The mortar strength was a critical factor for the compressive strength of the RCB masonry.

**Table 2:** Measured cracking load, failure load and compressive strength of specimens.

Specimen	Group A			Group B			Group C		
	cracking load *P*_c_ (kN)	failure load *P*_u_ (kN)	compressive strength *f*_c,m_ (MPa)	cracking load *P*_c_ (kN)	failure load *P*_u_ (kN)	compressive strength *f*_c,m_ (MPa)	cracking load *P*_c_ (kN)	failure load *P*_u_ (kN)	compressive strength *f*_c,m_ (MPa)
KYM *i*-1	139	215	2.12	145	225	2.22	-	-	-
KYM *i*-2	128	196	1.93	192	288	2.84	-	-	-
KYM *i*-3	148	224	2.21	165	255	2.51	-	-	-
KYM *i*-4	122	187	1.84	198	305	3.01	241	365	2.83
KYM *i*-5	144	224	2.21	133	204	2.01	231	346	2.89
KYM *i*-6	113	173	1.71	125	192	1.89	221	335	2.81
Average	132	203	2.00	160	245	2.41	231	348	2.84

#### 2.1.5. Compressive Strength Calculation

The compressive strength of common clay brick has been studied by some scholars locally and abroad. In general, brick strength and mortar strength are two critical factors that influence the compressive strength of brick masonry. Calculated equations have been proposed for brick masonry compressive strength based on the identification of theory and experience. The representative theoretical equations were presented by Francis [22], Hilsdorf [23] and Hendry [24]. The theoretical equations presented by Francis and Hilsdorf were deduced based on a prism piled up by laying bricks. It cannot reflect the elastoplastic performance of masonry material and remains to be further researched. Hendry has made some supplements to the above derivation method adopted by Francis and Hilsdorf. The biaxial compression-tension performance of brick and the triaxial compression performance of mortar were studied by Hendry to make up the deficiency of the assumption adopted by Francis and Hilsdorf. The representative empirical equations were presented by Oнищик [25] and Grimm [26]. The equation presented by Oнищик has taken into consideration the primary factors affecting the masonry compressive strength, but the calculation is relatively complicated with more parameters. Furthermore, considering the primary factors affecting the masonry compressive strength, the equation presented by Grimm had a great limitation and was only suitable for the calculation of brick masonry prism compressive strength. The equation adapted by British Standards [27] and ISO/TC179 for the calculation of masonry compressive strength was simpler by form, but did not match with reality. Compared with other equations widely used internationally, Equation (2) [16], as below, for the calculation of average compressive strength of masonry was adapted by the current design code of masonry structures (GB50003-2011) in China.

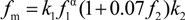
(2)
where *f*_m_ is the average compressive strength of masonry (MPa), *f*_1_ is the average compressive strength of the block (MPa), *f*_2_ is the average compressive strength of mortar, *k*_1_ is a parameter related to the type of block and construction method, *α* is a parameter related to the height of block and *k*_2_ is a correction coefficient of compressive strength related to the low-strength mortar, when *f*_2_ ≥ 1.0, *k*_2_ = 1.0. The parameters *k*_1_ = 0.78, α = 0.5 and *k*_2_ = 1.0 when calculating the average compressive strength of common clay brick masonry.

In this paper, we adopt Equation (2) and the parameters *k*_1_ = 0.55, α = 0.5 and *k*_2_ = 1.0 for the calculation of the RCB masonry average compressive strength, and the strength conversion coefficient ψ is related, as well. Table 3 compares the calculated values by Equation (2) with the measured values. It can be seen that the calculation results agree well with the experimental results. Equation (2) with appropriate parameters can be used to predict the average compressive strength of RCB masonry.

**Table 3:** Comparison of average compressive strength between calculated values and measured values.

Groups	*f*_1_ (MPa)	*f*_2_ (MPa)	Calculated Values (MPa)	Measured Values (MPa)	Errors (%)
A	7.90	4.84	2.12	2.00	5.98
B	7.90	7.33	2.40	2.41	0.60
C	7.90	9.44	2.63	2.84	7.41

#### 2.1.6. Elastic Modulus and Poisson’s Ratio

**Figure 4:**
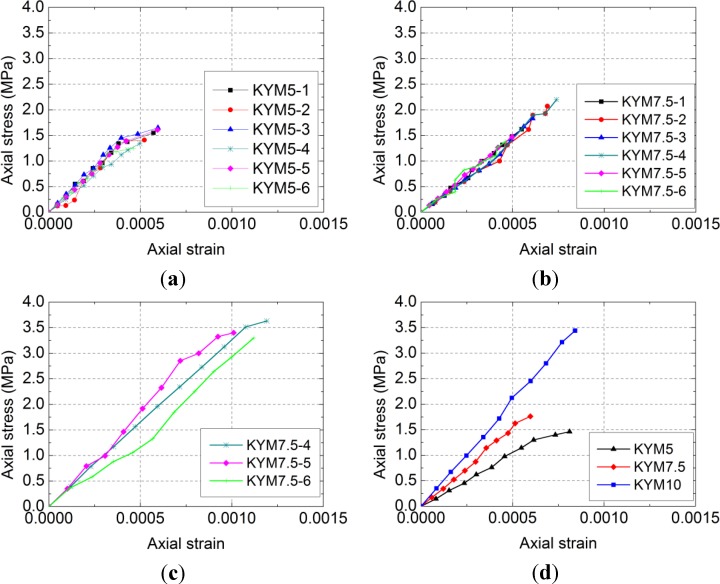
Measured axial stress-axial strain curves of different mortar strengths. (**a**) Group A; (**b**) Group B; (**c**) Group C; (**d**) Average stress-axial strain curves of different mortar strengths.

Figure 4 shows the measured axial stress-axial strain curves of different mortar strengths. It shows that the stress-strain curves were nearly linear before reaching the failure load. The compressive strength of the RCB masonry increased as the mortar strength increased.

The elastic modulus of RCB masonry takes the secant modulus, where the stress σ equals 0.4 *f*_m_. The measured elastic modulus *E* and Poisson’s ratio ν are listed in Table 4. Table 4 shows that the elastic modulus and Poisson’s ratio of the RCB masonry increase with the increase of the mortar strength.

**Table 4:** Elastic modulus and Poisson’s ratio.

Group	*f*_1_ (MPa)	*f*_2_ (MPa)	*f_m_* (MPa)	ν	*E* (MPa)
A	7.90	4.84	2.00	0.162	2761.5
B	7.90	7.33	2.41	0.176	3069.7
C	7.90	9.44	2.84	0.191	3320.4

### 2.2. Seismic Performance of RCB Masonry Walls

#### 2.2.1. Materials and Specimen Preparation

The measured tensile yield strength of the vertical reinforcement (hot-rolled ribbed steel bar and a diameter of 20 mm) and the U-shaped horizontal reinforcement (hot-rolled plain steel bar and a diameter of 4 mm) were 375 and 312 MPa, respectively. The measured prism compressive strength of fine aggregate concrete was13.4 MPa, while the measured compressive strength of RCB and mortar were 7.90 and 7.44 MPa, respectively.

Eight RCB masonry walls with height-to-width aspects ratios of 0.6 and 1.0 were fabricated and tested under reversed cyclic loading. The specimens can be divided into four groups according to the wall thickness and height-to-width aspects ratio. The variables involved included the wall-panel aspect ratio, wall thickness, distribution of vertical reinforcement and the axial compressive load. Specimen details are summarized in Table 5.

In the second column of Table 5, the letters MWA and MWB in the specimen labels stand for the masonry wall without vertical reinforcement and the masonry wall with vertical reinforcement, respectively. The number that follows (1, 2, 3, and 4) is the number of the group.

**Table 5:** Details of the specimens.

Group	Specimen	Height-to-Width Aspect Ratios	Wall Thickness (mm)	Number of Vertical Reinforcement	Axial Compressive Force (kN)
Group 1	MWA-1	0.6	260	0	348
	MWB-1	0.6	260	3	348
Group 2	MWA-2	1.0	260	0	245
	MWB-2	1.0	260	2	245
Group 3	MWA-3	0.6	390	0	522
	MWB-3	0.6	390	3	522
Group 4	MWA-4	1.0	390	0	367
	MWB-4	1.0	390	2	367

**Figure 5:**
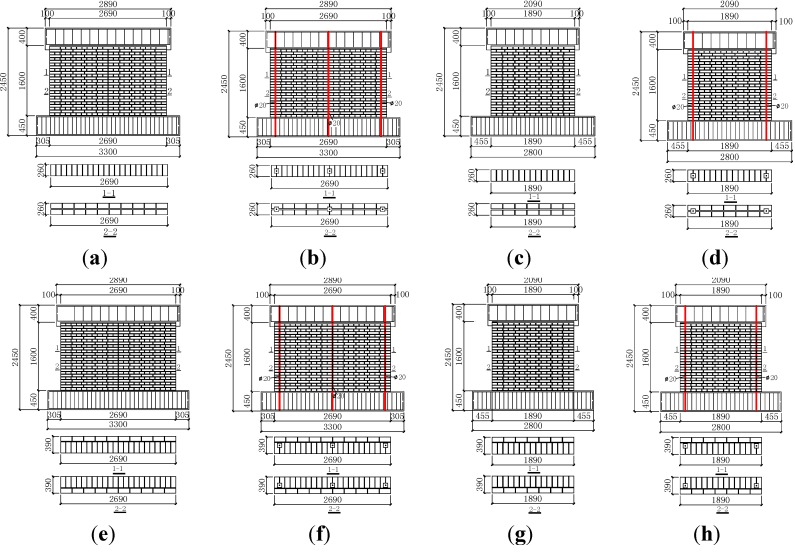
The dimensions of all of the specimens: (**a**) MWA-1; (**b**) MWB-1; (**c**) MWA-2; (**d**) MWB-2; (**e**) MWA-3; (**f**) MWB-3; (**g**) MWA-4; (**h**) MWB-4. (Unit: millimeters)

**Figure 6:**
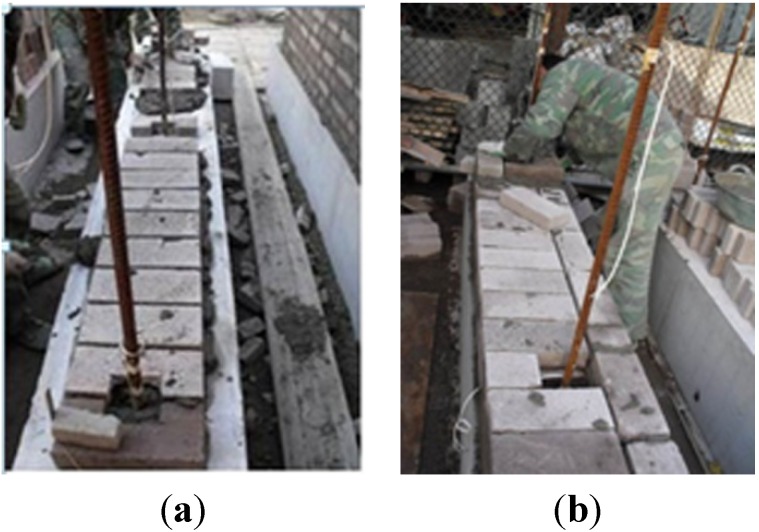
Settings of the vertical reinforcement: (**a**) Wall thickness of 260 mm; and (**b**) Wall thickness of 390 mm.

The size of RCB was 260 mm × 115 mm × 53 mm. The vertical reinforcement was arranged in the center of 100 mm × 100 mm square holes, which were formed by specially shaped bricks and distributed symmetrical on the cross-section of the wall. Fine aggregate concrete was cast into the square holes to form concealed columns. Moreover, vertical reinforcement was arranged at both ends of the wall as a priority and, then, in the center, when the wall was too long. Three vertical steel bars were set separately at both ends and the center of the specimens, MWB-1 and MWB-3. Two vertical steel bars were set separately at both sides of the specimens, MWB-2 and MWB-4. The distance from the centroid of vertical reinforcement to the nearby end edge of the wall was 130 mm. The roots of vertical steel bars were anchored into the base of specimens. The vertical reinforcement was connected with the wall by a U-shaped horizontal reinforcement every six brick units. Details of the geometry and reinforcement are shown in Figure 5. The settings of the vertical reinforcement are shown in Figure 6.

#### 2.2.2. Test Setup, Instrumentation and Procedure

**Figure 7:**
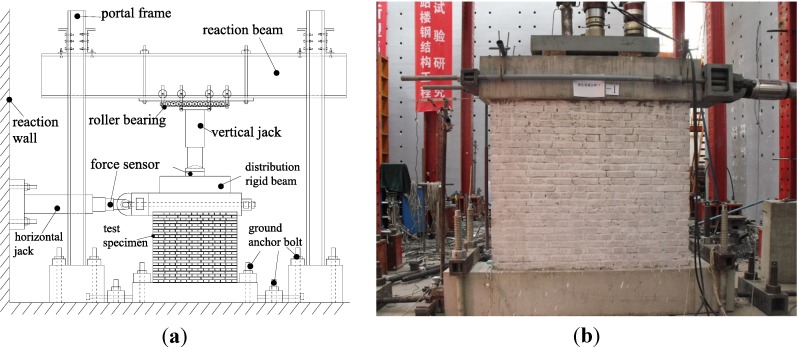
Test setup: (**a**) Schematic diagram; (**b**) Photograph.

The test setup is shown in Figure 7. The specimen was connected to the top rigid girder and the concrete base. Vertical loading was applied firstly through a 1000 kN jack above the rigid distribution girder and kept constant. Then, lateral load reversals were applied to the top rigid girder by a servo actuator, which was supported by the strong reaction wall in the laboratory.

The axial compression ratio in this experiment was taken as 0.2 for the specimens. Then, the applied vertical load was calculated by the following equation.

*N* = *nf_m_A* (3)

where *N* is the applied axial load, n is the experimental axial load ratio, *f*_m_ is the average measured compressive strength of the RCB masonry, *f*_m_ = 2.49 MPa, and *A* is the cross-sectional area of the RCB masonry wall.

In the test, both load and displacement controls were adopted at different loading stages. The load-control method was adopted at the early loading stage, while the displacement-control method was after the yield point. Force sensors were set at the end of the vertical jack and the horizontal jack to monitor the loads. A displacement sensor was set at the middle of the rigid girder to monitor the horizontal displacement. All strains, displacements and loads were recorded and analyzed with the IMP (Interface Message Processor) data gathering system connected to the specimens. The cracking process of the wall was monitored visually during the tests.

#### 2.2.3. Test Results

##### Failure Patterns

**Figure 8:**
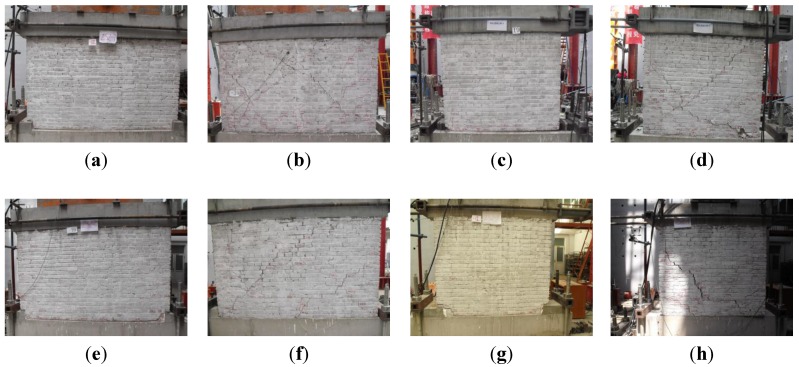
Crack patterns and failure of the specimens: (**a**) MWA-1; (**b**) MWB-1; (**c**) MWA-2; (**d**) MWB-2; (**e**) MWA-3; (**f**) MWB-3; (**g**) MWA-4; (**h**) MWB-4.

The crack patterns and failure patterns of all specimens are shown in Figure 8. For the specimens without vertical reinforcement, MWA-1, MWA-2, MWA-3 and MWA-4, similar failure patterns could be observed: the horizontal cracks appeared firstly in the horizontal mortar between the bottom of the specimen and the concrete base and, then, developed gradually through the whole cross-section. The specimens showed the characteristics of shear sliding, and the bottom corner of the specimen crushed. As for the specimens with vertical reinforcement, MWB-1, MWB-2, MWB-3 and MWB-4, the horizontal cracks also appeared firstly in the horizontal mortar between the bottom of the specimen and the concrete base. Then, inclined cracks appeared in the specimen with an X shape. The inclined cracks developed gradually with the increase of the horizontal load and through the wall. The failure model was the shear-compression failure. As for the specimens with three vertical steel bars, MWB-1 and MWB-3, compared to the specimens with two vertical steel bars, MWB-2 and MWB-4, the middle vertical steel bar limited the development of the inclined cracks. The entire wall was divided into two areas, and in each area appeared inclined cracks with an X shape.

##### Load-Bearing Capacity

Table 6 shows the cracking strength *F*_c_, the effective yielding strength *F*_y_ and the lateral ultimate strength *F*_max_ of the eight specimens; where *F*_c_ is the cracking load corresponding to the first occurrence of a crack appearing on the specimens and *F*_max_ is the lateral ultimate load-bearing capacity, which is the maximum lateral load applied for the specimens. *R*_c_, *R*_y_ and *R*_max_ represent the ratio of the cracking strength, the yield strength and the ultimate strength of the specimen with vertical reinforcement to the specimens without vertical reinforcement, respectively.

**Table 6:** Measured cracking strength,yield strength and ultimate strength.

Specimen	*F*_c_ (kN)	*R*_c_	*F*_y_ (kN)	*R*_y_	*F*_max_ (kN)	*R*_max_
MWA-1	82.22	1.2315	171.67	1.7698	220.87	1.7241
MWB-1	101.25		303.83		380.80	
MWA-2	60.61	1.5021	107.24	1.9788	131.00	1.9242
MWB-2	91.04		212.21		252.07	
MWA-3	89.20	1.6896	249.40	1.7190	326.53	1.5270
MWB-3	150.72		436.2		496.88	
MWA-4	84.35	1.1937	162.96	1.6000	195.55	1.5610
MWB-4	100.69		260.74		305.25	

It can be seen from Table 6 that the walls with vertical reinforcement (specimens MWB-1, MWB-2, MWB-3 and MWB-4) showed better load-bearing capacity than those without vertical reinforcement (specimens MWA-1, MWA-2, MWA-3 and MWA-4).

##### Deformation

**Figure 9:**
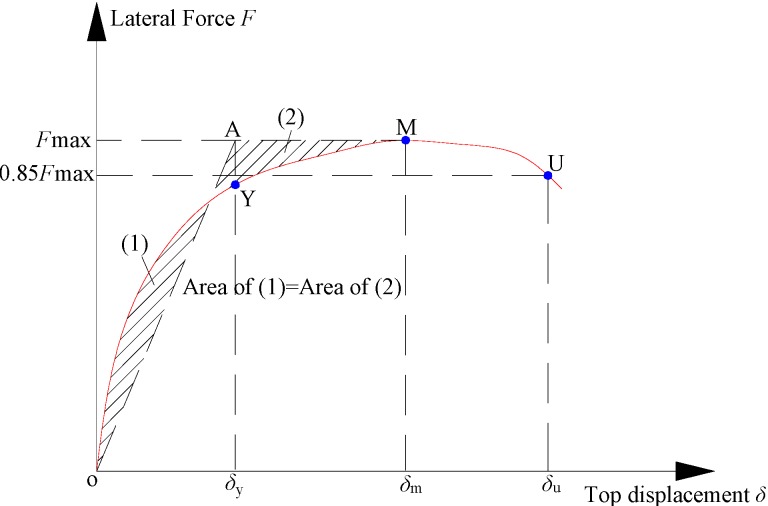
Determination of the yield and ultimate points.

The measured displacements and displacement drifts of each specimen are listed in Table 7. All of the displacements were measured at the middle of the rigid girder, as shown in Figure 8. The values in Table 7 are defined as: δ_y_ is the yielding displacement, θ_y_ is the yielding displacement drift corresponding to δ_y_, δ_u_ is the ultimate displacement and θ_u _ is the ultimate displacement drift corresponding to δ_u_. The yielding displacement δ_y_ in Table 7 is obtained by the equivalent yield point determined on the skeleton curve. The so-called “equivalent energy method” [28] was adopted to determine the equivalent yield point, and its basic principle is illustrated in Figure 9. The ultimate state was defined by the point on the descending section of the envelope curve with 15% force degradation.

**Table 7:** Measured yield displacement and ultimate displacement.

Specimen	δ_y_ (mm)			θ_y_ (%) Average	δ_u_ (mm)			θ_u_ (%) Average
	Negative	Positive	Average		Negative	Positive	Average	
MWA-1	3.74	2.24	2.99	0.19	8.35	8.75	8.55	0.48
MWB-1	5.44	4.19	4.82	0.30	15.19	21.18	18.19	1.02
MWA-2	5.25	2.70	3.98	0.24	8.47	10.41	9.44	0.53
MWB-2	7.16	4.16	5.66	0.35	12.15	16.74	14.45	0.81
MWA-3	4.38	4.85	4.62	0.29	12.78	13.55	13.17	0.74
MWB-3	6.89	6.15	6.52	0.41	16.59	21.25	18.92	1.06
MWA-4	5.85	3.39	4.62	0.29	14.52	16.46	15.49	0.87
MWB-4	4.79	7.41	6.10	0.38	20.54	23.76	22.15	1.12

The results in Table 7 showed that compared to the specimens without vertical reinforcement, MWA-1, MWA-2, MWA-3 and MWA-4, the yielding displacement δ_y_ of the specimens with vertical reinforcement, MWB-1, MWB-2, MWB-3 and MWB-4, increased by 61%, 42%, 41% and 32%, respectively. The ultimate displacement δ_u_ of the specimens with vertical reinforcement, MWB-1, MWB-2, MWB-3 and MWB-4, increased by 112%, 53%, 43% and 43%, respectively. The walls with vertical reinforcement showed better deformation capacity than those without vertical reinforcement.

#### 2.2.4. Hysteretic Loops and Skeleton Curves

**Figure 10:**
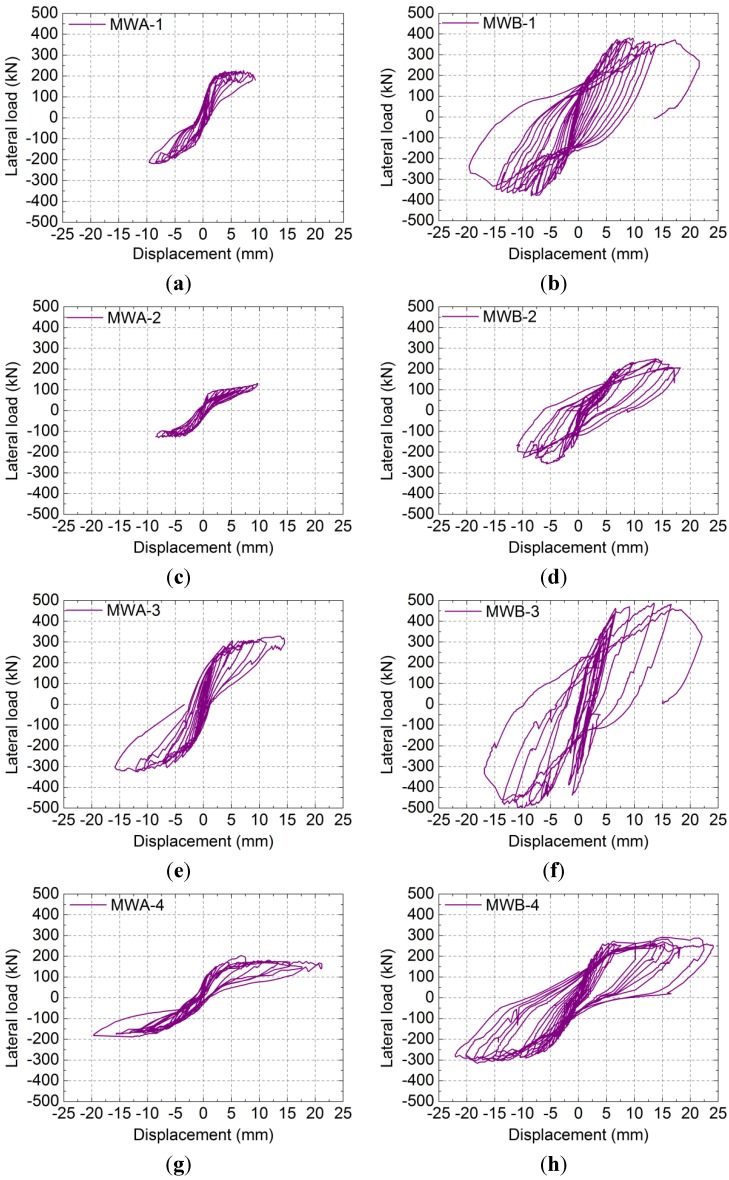
Lateral load-displacement hysteretic loops of specimens: (**a**) MWA-1; (**b**) MWB-1; (**c**) MWA-2; (**d**) MWB-2; (**e**) MWA-3; (**f**) MWB-3; (**g**) MWA-4; (**h**) MWB-4.

**Figure 11:**
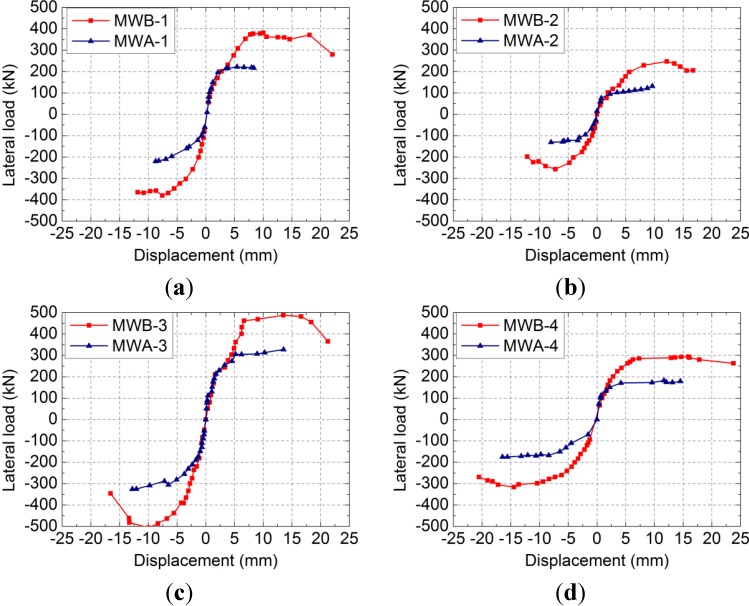
Lateral load-displacement skeleton curves of specimens: (**a**) MWA-1 and MWB-1; (**b**) MWA-2 and MWB-2; (**c**) MWA-3 and MWB-3; (**d**) MWA-4 and MWB-4.

The measured lateral load-displacement hysteretic loops and skeleton curves of the specimens are shown in Figure 10 and Figure 11, respectively; where, δ is the displacement measured at the middle of the rigid girder and *F* is the lateral load. As shown in Figure 10 and Figure 11: Compared to the specimens MWA-1, MWA-2, MWA-3 and MWA-4, the specimens with vertical reinforcement, MWB-1, MWB-2, MWB-3 and MWB-4, have the advantages of fuller hysteretic loops, slower stiffness degradation and higher elastic-plastic deformation ability.

#### 2.2.5. Energy Dissipation

The energy dissipation values were calculated by taking the average area surrounded by the positive and negative of the skeleton curve and the horizontal axis. The measured energy dissipation values are listed in Table 8, where *A* is the energy dissipation of the specimen and *R*_A_ is the ratio of the energy dissipation between the specimen with vertical reinforcement and the specimen without vertical reinforcement.

The results in Table 8 show that the measured energy dissipation capacity values of MWB-1, MWB-2, MWB-3 and MWB-4 are higher by 274%, 174%, 113% and 139%, respectively. This shows that the energy dissipation capacities of the specimens with vertical reinforcement are significantly greater than the specimens without vertical reinforcement.

**Table 8:** Experimental results of energy dissipation.

Specimen	*A* (kN∙mm)	*R*_A_
MWA-1	3,054.91	3.74
MWB-1	11,436.13	
MWA-2	2,042.21	2.74
MWB-2	5,611.24	
MWA-3	7,091.88	2.12
MWB-3	15,102.86	
MWA-4	4,712.14	2.39
MWB-4	11,274.29	

#### 2.2.6. Lateral Ultimate Strength Calculation

The lateral ultimate strength of the RCB masonry wall with vertical reinforcement contains two parts. One is the contribution of the RCB wall without vertical reinforcement; the other is the contribution of concealed concrete columns. The shear strength equation of RCB walls embedded with vertical reinforcement is proposed as below and refers to the existing shear bearing capacity calculation equation, which is given by the design code of masonry structures, GB 5003-2011 [29].

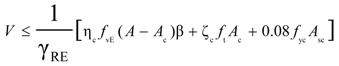
(4)
where γ_RE_ is the anti-seismic adjusting factor of the bearing capacity, which reflects the influences of the concealed columns at the end of the wall section to the bearing capacity. *f*_vE_ is the shear strength of RCB masonry fractured along the ladder section. *A* is the total cross-section area of the wall. *A*_c_ is the cross-section area of the midst concealed concrete column. *A*_sc_ is the total cross-section area of the reinforcement in the middle of the concealed concrete column. *f*_t_ is the concrete uniaxial tensile strength. *f*_yc_ is the tensile strength of the vertical reinforcement. η_c_ is the constraint correction coefficient of the wall, which reflects the influences of the concealed columns in the middle of the wall section to the bearing capacity. ζ_c_ is the contribution coefficient of the middle concealed concrete column. β is the influence coefficient of the RCB masonry, considering the aspect ratio λ, β = 1.3 − 0.5λ (0.6 ≤ *λ* ≤ 1.0). As for the RCB masonry without vertical reinforcement, *A*_c_ = 0, η_c_ = 1, *A*_sc_ = 0.

The calculation of the lateral ultimate strength of eight specimens according to Equation (4) is shown in Table 9. It can be seen that the calculation results agree well with the experimental results. The proposed equation can be used to predict the lateral ultimate strength of the RCB masonry wall with vertical reinforcement.

**Table 9:** The calculated values and measured values of lateral ultimate strength.

Specimens	Calculated (kN)	Measured (kN)	Errors (%)
MWA-1	233.25	220.87	5.61
MWB-1	374.16	380.80	1.74
MWA-2	131.11	131.00	0.08
MWB-2	237.17	252.07	5.91
MWA-3	349.87	326.53	7.14
MWB-3	537.25	496.88	8.12
MWA-4	196.65	195.55	0.56
MWB-4	348.83	325.25	7.25

## 3. FEM Analysis

### 3.1. Material Constitutive Models

The damage plasticity model from the ABAQUS software was applied to simulate the concrete constitutive law. The stress-logarithmic strain relationship of concrete in code GB50010-2010 [30] was used here. The test values were obtained by the concrete cylinder compression test, and Poisson’s ratio was taken as 0.2.

**Figure 12:**
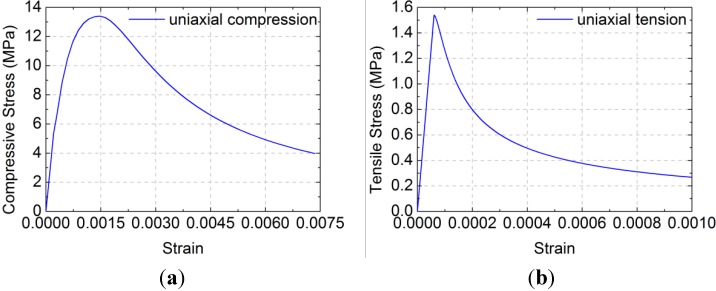
Constitutive law of concrete under uniaxial compression: (**a**) Compressive stress-strain curve; (**b**) Tensile stress-strain curve.

The stress-strain curve of a standard concrete cylinder subjected to a uniaxial compression is solved mathematically by Equation (5):

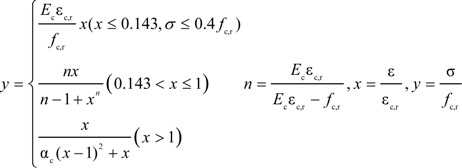
(5)
where α_c_ is the coefficient at the descent stage of the uniaxial compression stress-strain curve of concrete, *f*_c,r_ is the representative value of uniaxial compressive strength of concrete, *ε*_c,r_ is the peak compressive strain of concrete corresponding to *f*_c,r_ and *E*_c_ is the elastic modulus of concrete. In the numerical simulation, α_c_ = 0.74, *f*_c,r_ = 13.4 MPa, ε_c,r_ = 0.00133 and *E*_c_ = 2.55 × 10^4^ MPa are based on the test concrete compressive strength and elastic modulus. The compressive stress-strain curve of concrete is plotted in Figure 12a.

The compression damage factor *d*_c_ can be calculated by Equation (6):

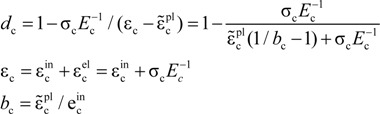
(6)
where is 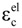 the elastic compression strain, 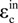 is the inelastic compression strain and 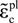 is the plastic compression strain. In this paper, *b*_c_ = 0.7.

The “σ-ε” formula for tension is given in the Equation (7):

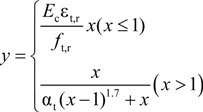
(7)
where *α*_t_ is the coefficient at the descent stage of the uniaxial tension stress-strain curve of concrete, *f*_t,r_ is the representative value of uniaxial tension strength of concrete and ε_t,r_ is the peak tension strain of concrete corresponding to *f*_t,r_. In the numerical simulation, α_t_ = 0.7399, *f*_c,r_ = 1.54 MPa and ε_c,r_ = 0.000604 are based on the test concrete tensile strength and elastic modulus. The tensile stress-strain curve of concrete is plotted in Figure 12b.

The calculation of tension damage factor *d*_t_ is given in Equation (8):

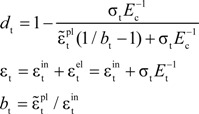
(8)
where, 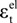 is the elastic compression strain, 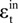 is the inelastic tension strain and 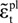 is the plastic tension strain. In this paper, *b*_t_ = 0.1.

The constitutive law of the RCB wall: Integrated modeling by the plastic-damage model was based on ABAQUS. The compressive strain-stress curve of RCB masonry obtained was based on Yang’s model [31]. The true values were obtained by the compression test on the RCB masonry and Poisson’s ratio was taken as 0.15.

**Figure 13:**
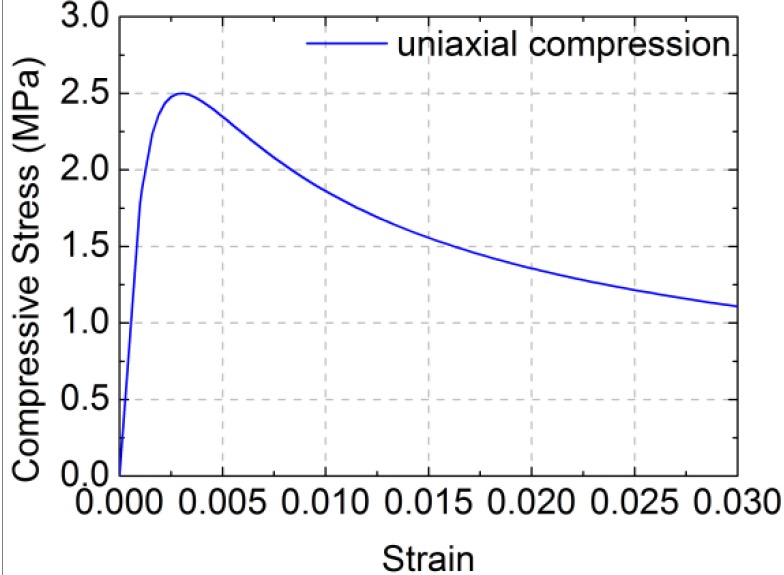
Constitutive law of RCB masonry under uniaxial compression.

The “σ_cm_-ε_cm_” formula for the compression of RCB masonry is given in Equation (9):

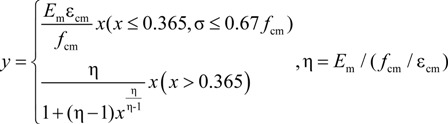
(9)
where *f*_cm_ is the representative value of the uniaxial compressive strength of RCB masonry, ε_cm_ is the peak compressive strain corresponding to *f*_cm_ and *E*_m_ is the elastic modulus of RCB masonry. In the numerical simulation, *f*_cm_ = 2.49 MPa, ε_cm_ = 0.003 and *E*_m_ = 2400 MPa are based on the test masonry strength and elastic modulus. The constitutive low of RCB masonry under uniaxial compression is shown in Figure 13.

**Figure 14:**
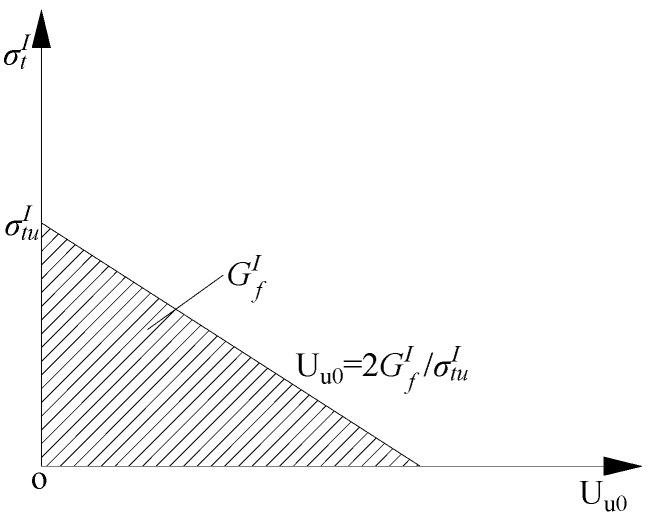
Post-failure stress-fracture energy curve in the descent stage for RCB masonry.

The tensile softening uses the method based on fracture energy; where 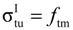, *f*_tm_ is the uniaxial tension strength of the RCB masonry, 
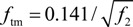
 [16] and *f*_2_ is the compressive strength of the mortar. The fracture energy 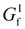 was taken as 20 N·m^−^^1^ [32]. The post-failure stress-fracture energy curve in the descent stage is shown in Figure 14.

**Figure 15:**
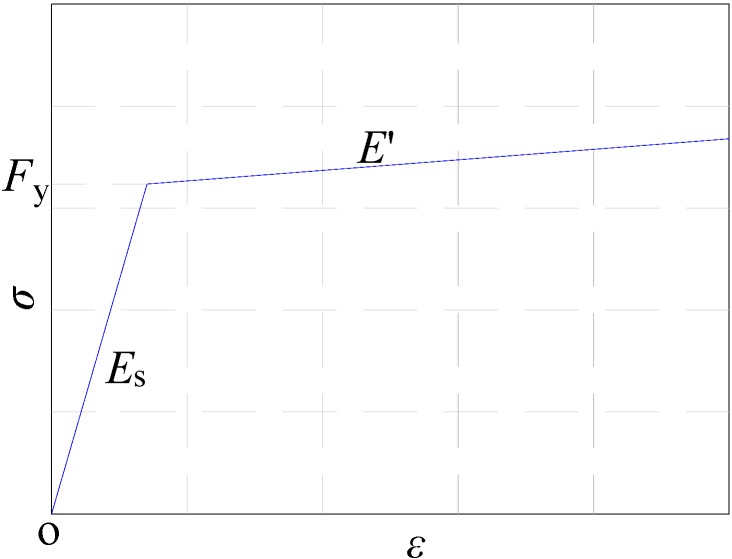
Stress-strain curve of reinforcement.

The “plasticity” model from ABAQUS software was adopted to simulate the reinforcement law. The stress-strain curve is plotted in Figure 15. Each stress-strain curve, made up of two linear portions, represents the character of a bare mild steel bar; where the modulus *E*_s_ is the elastic modulus of the reinforcement and the modulus *E′* is the deformation modulus at the strain hardening stage, *E′* = 0.01*E*_s_. The test yield strength and elastic modulus of the reinforcement were adopted in the paper, and Poisson’s ratio was taken as 0.3.

### 3.2. Finite Element Analysis Model

**Figure 16:**
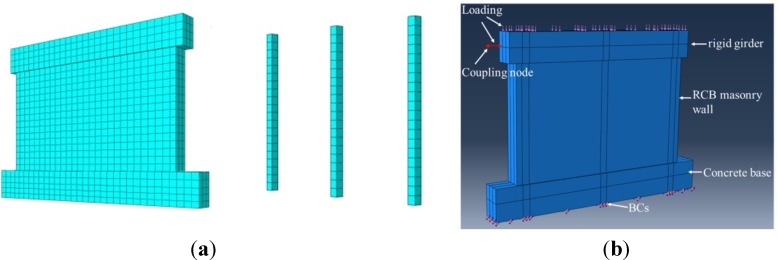
Finite element analysis model of MWB-1: (**a**) Element mesh; (**b**) Loading and boundary conditions (BCs).

Solid element C3D8R was used for concrete and masonry wall, and truss element T3D2 was used for reinforcement. All of the interface models, including the wall to load beam, wall to base and concealed concrete column to wall, were composed by contact in the lateral axis and stick-slip along the tangential axis. The “hard contact” model was adopted for the contact on the lateral axis and the “Coulomb friction” model for the stick-slip along the tangential axis. The friction coefficient was taken as 0.7 according to the code GB5003-2011[29]. Reinforcements were all embedded in the concrete. The bottom of the base was fixed rigidly. A vertically distributed load was applied to the top of the rigid girder first and kept constant. Then, horizontal displacement was applied to a coupling node in the middle plane of the rigid girder. A typical finite element analysis model of MWB-1 is shown in Figure 16.

### 3.3. Finite Element Simulation Results and Analysis

**Figure 17:**
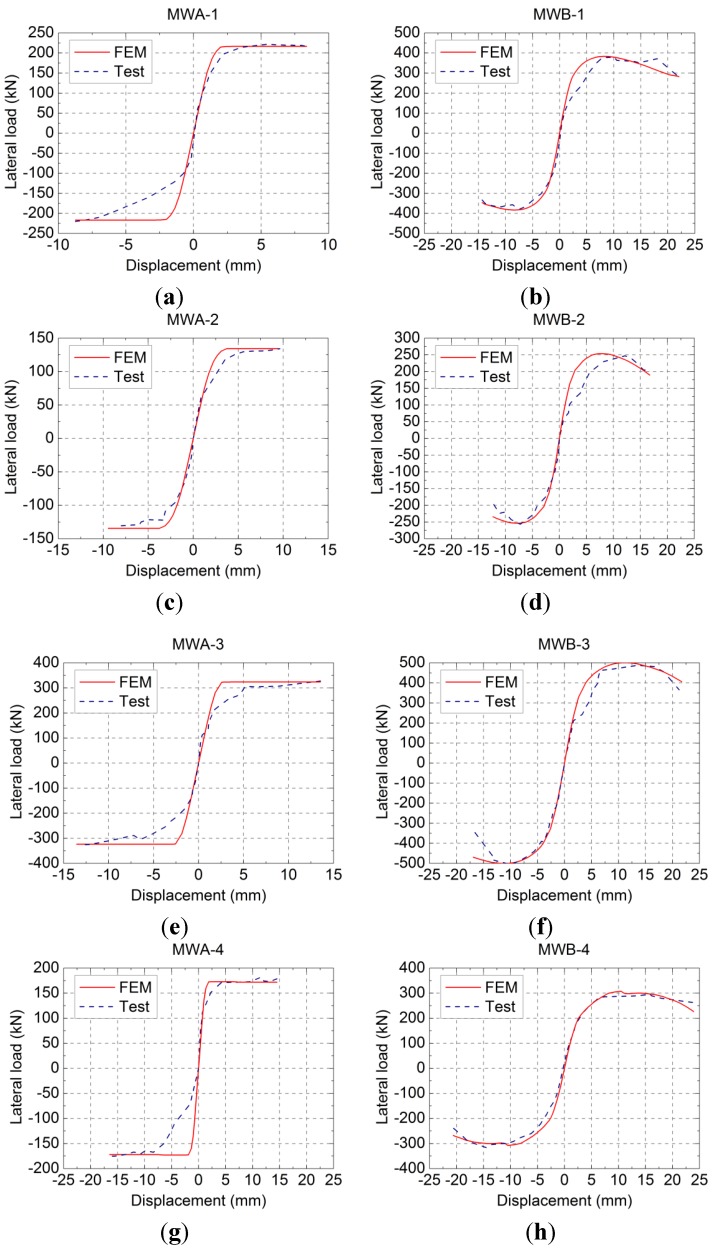
Comparison of lateral load *-*displacement skeleton curves between calculated values and measured values: (**a**) MWA-1; (**b**) MWB-1; (**c**) MWA-2; (**d**) MWB-2; (**e**) MWA-3; (**f**) MWB-3; (**g**) MWA-4; (**h**) MWB-4.

Figure 17 shows the comparison of lateral load*-*displacement skeleton curves between calculated values and measured values. It shows that the initial elastic stage of the calculated curves is in good agreement with that of the measured curves. Because of the complex damage process of masonry walls and the sliding, the relative error becomes higher in the elastic-plastic stage. However, the overall trend and the bearing capacity are still in good agreement with the measured values.

### 3.4. Effect of Axial Compressive Stress *Σy* on Lateral Load-Displacement Curves and Lateral Ultimate Strength

**Figure 18:**
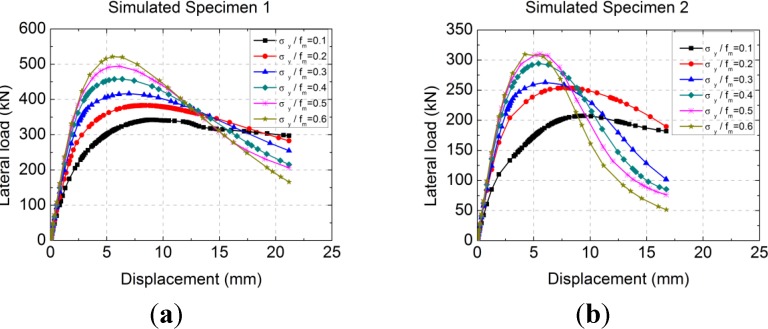
The calculated lateral load-displacement curves under different σ_y_/*f*_m_: (**a**) Simulated Specimen 1; (**b**) Simulated Specimen 2.

The mechanical performances of two simulated specimens, namely simulated Specimen 1 and simulated Specimen 2, were analyzed under six compressive stress ratios by ABAQUS. The parameter of the simulated Specimen 1 was the same as the test specimen MWB-1 with an aspect ratio of 0.6, and the simulated Specimen 2 was the same as the test specimen MWB-2 with an aspect ratio of 1.0. The change rules of lateral load-displacement curves to axial compressive stress ratio σ_y_/*f*_m_ are analyzed, where σ_y_ is the axial compressive stress of the wall section and *f*_m_ is the average compressive strength of RCB masonry. The calculated lateral load-displacement curves of the two simulation specimens under six compressive stress ratios are shown in Figure 18.

**Figure 19:**
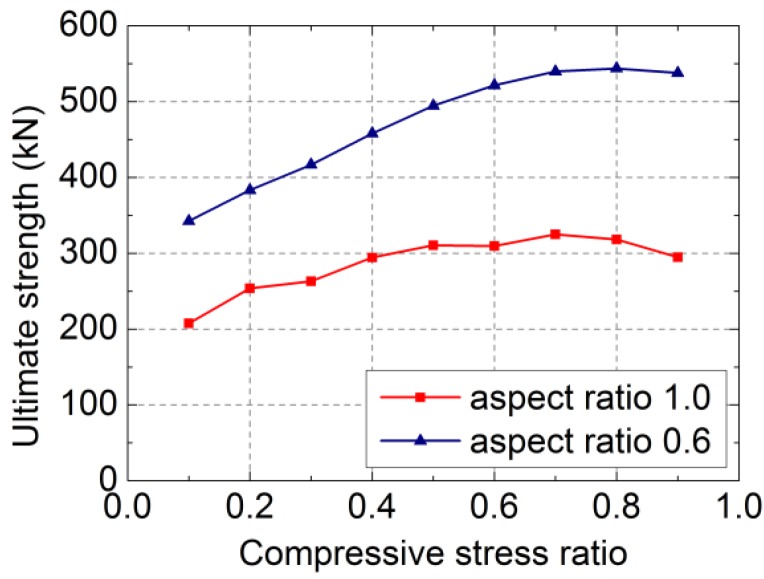
The concluded ultimate strength (*F*_max_)-compressive stress ratio (σ_y_/*f*_m_) curves.

Figure 18 shows that under a certain limit of compressive stress ratio, the lateral ultimate strength (*F*_max_) of the RCB masonry walls with vertical reinforcement grows with the increase of the compressive stress ratio by a slow growth rate. However, the ductility declines with the increase of the compressive stress ratio by a fast degradation rate. The compressive stress ratio has little effect on the initial elastic stiffness, but affects the stiffness degradation speed gradually during the elastic stage to the elastic-plastic stage. The concluded ultimate strength (*F*_max_)-compressive stress ratio (σ_y_/*f*_m_) curves of the two simulation specimens under nine compressive stress ratios are shown in Figure 19.

Figure 19 shows that the lateral ultimate strength of the RCB wall with vertical reinforcement experiences the process from the increase stage to the decrease stage with the increase of the compressive stress ratio. As for the two simulated specimens, the ultimate strength increases with the increase of the compressive stress ratio when the compressive stress ratio is less than 0.7, but declines when the compressive stress ratio exceeds 0.7.

## 4. Conclusions

Based on the tests and the analytical results, the following conclusions can be drawn:

1. The failure patterns of the RCB masonry are similar to common clay brick masonry when subjected to axial compression. The elastic modulus and Poisson’s ratio of the RCB masonry increase gradually as the mortar strength increases.
2. For the RCB masonry walls with vertical reinforcement, the concealed concrete columns are effective in restricting the deformations and cracks of the wall and inducing the failure patterns to transform from brittleness to ductility. Compared to the RCB masonry walls without vertical reinforcement, the RCB masonry walls with vertical reinforcement have the advantages of fuller hysteretic loops, a lighter pinch in the middle of the loops, slower stiffness degradation and stronger elastic-plastic deformation ability.
3. Under a certain limit of the compressive stress ratio, the lateral ultimate strength of the vertical reinforcement RCB masonry walls grows with the increase of the compressive stress ratio by a slow growth rate. However, the ductility declines by a fast degradation rate. The compressive stress ratio has little effect on the initial elastic stiffness, but affects the stiffness degradation speed gradually during the period from the elastic stage to the elastic-plastic stage.
4. The RCB masonry walls with vertical reinforcement have the advantages of easy construction, lower cost and good seismic performance, so they can be a good choice for the seismic design of rural low-rise buildings.
